# Synthesis and Characterization of *N*-Isopropylacrylamide Microspheres as pH Sensors

**DOI:** 10.3390/s21196493

**Published:** 2021-09-29

**Authors:** Barry K. Lavine, Necati Kaval, Leah Oxenford, Mariya Kim, Kaushalya Sharma Dahal, Nuwan Perera, Rudolf Seitz, James T. Moulton, Richard A. Bunce

**Affiliations:** 1 Department of Chemistry, Oklahoma State University, Stillwater, OK 74078, USA; kavaln@ucmail.uc.edu (N.K.); loxenfor@bechtel.com (L.O.); mariak3d@gmail.com (M.K.); kaushalyadahal@gmail.com (K.S.D.); jtmoult@okstate.edu (J.T.M.); richard.a.bunce@okstate.edu (R.A.B.); 2 Department of Chemistry and Physics, Western Carolina University, Cullowhee, NC 28723, USA; uperera@email.wcu.edu; 3 Department of Chemistry, University of New Hampshire, Durham, NH 03824, USA; Rudi.Seitz@unh.edu

**Keywords:** *N*-isopropylacrylamide, nonionic polymer swelling, optical pH sensing, turbidimetry, thermodynamics of pH-induced polymer swelling

## Abstract

Swellable polymer microspheres that respond to pH were prepared by free radical dispersion polymerization using *N*-isopropylacrylamide (NIPA), *N*,*N*^′^-methylenebisacrylamide (MBA), 2,2-dimethoxy-2-phenylacetylphenone, *N*-tert-butylacrylamide (NTBA), and a pH-sensitive functional comonomer (acrylic acid, methacrylic acid, ethacrylic acid, or propacrylic acid). The diameter of the microspheres was between 0.5 and 1.0 μm. These microspheres were cast into hydrogel membranes prepared by mixing the pH-sensitive swellable polymer particles with aqueous polyvinyl alcohol (PVA) solutions followed by crosslinking with glutaric dialdehyde for use as pH sensors. Large changes in the turbidity of the PVA membrane were observed as the pH of the buffer solution in contact with the membrane was varied. These changes were monitored by UV–visible absorbance spectroscopy. Polymer swelling of many NIPA copolymers was reversible and independent of the ionic strength of the buffer solution in contact with the membrane. Both the degree of swelling and the apparent pK_a_ of the polymer microspheres increased with temperature. Furthermore, the apparent pK_a_ of the polymer particles could be tuned to respond sharply to pH in a broad range (pH 4.0–7.0) by varying the amount of crosslinker (MBA) and transition temperature modifier (NTBA), and the amount, pK_a_, and hydrophobicity of the pH-sensitive functional comonomer (alkyl acrylic acid) used in the formulation. Potential applications of these polymer particles include fiber optic pH sensing where the pH-sensitive material can be immobilized on the distol end of an optical fiber.

## 1. Introduction

pH is one of the most common laboratory measurements made due to the fact that many chemical and biological reactions depend on the control of pH. Because of this, many different methods exist for the measurement of pH ranging from colorimetric indicators to glass and metal electrodes. Although the glass electrode is the standard approach for measuring pH [1], the size of the electrode and the need for continued recalibration prevent it from being used in a variety of environmental and biomedical analysis problems, e.g., monitoring the rising acidity levels of oceans because ocean water is becoming enriched in carbon dioxide due to global warming [2], monitoring and controlling pH in fermentation baths [3], continuous in vivo pH measurements of blood in arteries and muscles for patients suffering from tissue ischemia [4], and pH monitoring of gastro-esophageal reflux disease, a digestive disorder related to the retrograde movement of gastric acid in the esophagus [5].

Optical pH sensing implemented with fiber optics [6,7,8,9] has been previously investigated as an alternative to a glass electrode for biomedical measurements because it combines small size and calibration stability. A pH indicator is immobilized at the distal end of an optical fiber by adsorption, covalent bonding, or entrapment. Interaction of the indicator with the sample solution leads to a change in its optical properties which is detected through the optical fiber by absorbance or fluorescence of the indicator. However, these types of measurements are subject to two limitations. Because they require photoexcitation of an indicator molecule, they are necessarily subject to some degree of photodegradation. As the sensing element becomes smaller, more intensity is required to get a measurable optical signal causing the rate of photodegradation to increase. Another limitation is that the measurement has to be made at the wavelength where the dye absorbs and/or emits which is usually in the visible region of the spectrum. As a result, fiber optic chemical sensors based on absorbance or luminescence cannot take full advantage of technology developed for fiber optic communications which involves measurements in the near infrared region of the spectrum. 

In order to overcome these drawbacks, swellable polymer particles that respond to pH have been investigated [10,11,12,13,14,15,16]. Although there is an extensive published literature on polymer swelling, there are only a few studies that have been performed to exploit this phenomenon for pH sensing despite the obvious advantages offered by polymer swelling as a transduction mechanism. One advantage is that the response involves a thermally stable polymer that does not degrade with time as compared to an indicator that is subject to photodegradation. Another advantage is that sensing is not restricted to wavelengths where the indicator dye absorbs or emits. Instead, sensing can be performed at any wavelength including the near infrared at which optical fibers have minimal attenuation. This reduces the cost of the required instrumentation and allows for remote measurements of pH several kilometers away where the measuring instrument is located. However, the lifetime of these sensors is limited by delamination of the swellable polymer. Swelling and shrinking introduces a shear force at the sensor/polymer interface that eventually breaks the covalent bonds that hold the polymer layer onto the substrate. This is a problem for any sensor configuration that involves direct immobilization of a swellable layer on a rigid substrate. 

For these reasons, a different approach to polymer swelling was taken in this study. Swellable polymer microspheres that are pH sensitive were suspended in a hydrogel. The idea of suspending swellable polymer microspheres in a hydrogel was first demonstrated by Seitz and Rooney [17], who synthesized pH-sensitive animated polystyrene microspheres suspended in a solution of hydroxyethylmethacrylate, which was then polymerized to form a hydrogel. In this study, lightly crosslinked pH-sensitive swellable polymer microspheres (approximately 1 μm in diameter as determined from the scanning electron micrograph snapshot) of *N*-isopropylacrylamide (NIPA) and alkyl acrylic acid were synthesized and investigated as a pH-sensitive indicator phase. The polymer particles were embedded in a polyvinlyalcohol (PVA) membrane. The change in the turbidity of the PVA membrane that occurs as a result of the change in the refractive index of the microspheres that accompany swelling and shrinking is measured. The microspheres swell and shrink with changing hydrogen ion concentration in the aqueous solution that is in direct contact with the PVA membrane. When the membrane is exposed to an alkaline solution, the microspheres swell due to deprotonation of the carboxylic acid group which causes the NIPA polymer solvent interaction parameter to decrease and the water content of the polyNIPA microspheres to increase. This (in turn) reduces the refractive index of the polyNIPA particles, so they are closer to the refractive index of the PVA hydrogel (which is 90% water), leading to a decrease in the amount of light reflected and a lower membrane turbidity as the percentage of the light reflected at the interface decreases as the difference in the refractive index between the two media (PVA membrane and polyNIPA particles) also decreases. 

Polymer swelling as implemented in this study using hydrogel membranes that contain swellable polymer particles sensitized to pH offers several important advantages for chemical sensing. The problem of delamination that occurs when a swellable polymer is directly immobilized on a surface is circumvented. Swelling and shrinking of the microspheres in the membrane has a minimal effect on the size of the PVA membrane and does not generate sufficient shear force to affect adhesion of the hydrogel to a substrate. The polymer microspheres swell freely in all three dimensions, increasing the volume change due to swelling and circumventing the problem of delamination that occurs when a swellable polymer is directly immobilized on a surface. The hydrogel membrane not only serves as a flexible medium to hold the microspheres in place but also acts as a “filter” to protect the microspheres from sample components such as humic acid or suspended particles that are too large to diffuse through the hydrogel. The polymer microspheres do not leach out of the membrane as compared to reagent phases. These membranes and the particles entrapped in them have been shown to be stable for several years. Based on previously published work on sensors that utilize swellable polymers [18], we deem this approach to be the most practical for the preparation of chemical sensors that will find applications to real world problems. 

In this study, swellable polymer microspheres that respond to pH were prepared by free radical dispersion polymerization using *N*-isopropylacrylamide (NIPA), *N*,*N*-methylenebisacrylamide (MBA), 2,2-dimethoxy-2-phenyl-acetophenone (DMPA), *N*-*tert*-butylacrylamide (NTBA), and a pH-sensitive comonomer (acrylic acid, methacrylic acid, ethacrylic acid, and propacrylic acid). The diameter of the microspheres synthesized was between 0.5 and 1 μm (as determined by the scanning electron micrograph snapshot of these polymer particles). The NIPA polymer microspheres were cast into hydrogel membranes prepared by mixing the pH-sensitive polyNIPA particles with aqueous polyvinyl alcohol (PVA) solutions followed by crosslinking with glutaric dialdehyde for use as pH sensors. Large changes in the turbidity of the PVA membrane were observed as the pH of the buffer solution in contact with the membrane was varied. These changes were monitored using UV–visible absorbance spectroscopy. Polymer swelling of the NIPA copolymers was generally reversible and independent of the ionic strength of the buffer solution in contact with the membrane. Both the degree of swelling and the apparent pK_a_ of the polymer particles increased with temperature. Previously published studies on pH-sensitive swellable polymers for optical sensing have focused on charged polymers [19,20]. For these polymers, an increase in the ionic strength of the buffer solution causes a reduction in the swelling. By comparison, acrylamide polymers have been previously investigated as materials suitable for chemical sensing [21]. The ease of polymerization and the wide range of functionalization possibilities through copolymerization using a limited number of monomers to impart new properties such as enhanced swelling or analyte specificity make acrylamides such as NIPA an ideal starting material for the construction of different chemical sensor platforms. Incorporation of even a small amount of functional co-monomer such as methacrylic acid allows NIPA-based polymers to respond to changes in pH. Although a large number of studies have appeared in the literature on pH initiated swelling of acrylamide microgels [22], most of these studies have been limited to a specific pH-sensitive functional comonomer or a narrow temperature range. Only a few studies have been undertaken to delineate the effects of temperature and ionic strength on the pH response of a series of NIPA copolymers containing different pH-sensitive functional co-monomers [23,24]. However, the formulations used to synthesize these NIPA copolymers contained the same amount of pH-sensitive comonomer, NTBA, and crosslinker. For this reason, it was necessary to undertake a more systematic investigation of the changes in the composition of the polymer formulation used and how these changes impact on the pH response of the NIPA polymer microspheres. The results of this study unequivocally demonstrate that the apparent pK_a_ of the NIPA polymer particles can be tuned by varying the quantity of the crosslinker (MBA), transition temperature modifier (NTBA), and the amount, pK_a_, and hydrophobicity of the pH-sensitive functional comonomer (alkyl acrylic acid) in the formulation.

## 2. Materials and Methods

### 2.1. Materials

NIPA and acetonitrile were obtained from ACROS Chemicals (Morris Plains, New Jersey, USA) and were used as received. Acrylic acid (AA), methacrylic acid (MAA), MBA, NTBA, DMPA, and PVA (MWT 85,000–146,000, 98–99% hydrolyzed) were obtained from Aldrich Chemical Co. (Milwaukee, WI, USA) and also were used as received. Glutaric dialdehyde (50% *w*/*w* solution in water) was also purchased from Aldrich and a 10% *w*/*w* solution was prepared by diluting 0.8848 g of the 50% *w*/*w* solution with deionized (DI) water. Ethacrylic acid (EAA) and propacrylic acid (PAA) were prepared using a procedure previously developed by Tirrell and coworkers [25]. Sodium hydroxide, hydrochloric acid, and acetic acid were purchased from Thermo-Fisher (Waltham, MA, USA), whereas sodium chloroacetate, 2-(*N*-morpholino)ethanesulfonic acid (MES) and 3-(*N*-morpholino)propanesulfonic acid (MOPS) were purchased from Sigma-Aldrich (Atlanta, GA, USA). 

### 2.2. Preparation of Buffer Solutions

Buffers were prepared using chloroacetic acid/sodium chloroacetate (pH 3.0–3.8), acetic acid (pH 3.9–5.4), MES (5.5–7.3) and MOPS (7.4 to 8) to cover the pH range 3–8. To prepare buffer solutions with different ionic strengths, a known amount of NaCl was added to 0.05 M buffer solutions. An on-line buffer calculator [26] was used to determine the composition of each buffer for a specific pH and ionic strength. All buffers were prepared using DI water obtained from a Corning Mega Pure distillation apparatus (Corning, NY, USA). The pH of each buffer was verified using a pH meter (Orion model 420A).

### 2.3. Synthesis of pH-Sensitive N-Isopropylacrylamide Copolymers

PolyNIPA particles that are pH sensitive were synthesized by photo-initiated free radical dispersion polymerization. The formulations used to prepare the pH-sensitive swellable polymer particles consisted of NIPA, 2-alkyl-acrylic acid (AA, MAA, EAA, or PAA), MBA, NTBA, and DMPA (photo radical initiator). NIPA, MBA, and NTBA were added to a 500 mL 3-necked round-bottomed Pyrex flask containing 100 mL acetonitrile. (Acetonitrile was selected as the solvent for the reaction as it defined the solubility threshold and the size of the colloidal polymer particles formed.) The monomer solution was stirred for 30 min in a closed system to dissolve all components while preventing oxygen from infiltrating into the reaction mixture. After 30 min, 0.2 g of DMPA and the alkyl acrylic acid comonomer were added to the 500 mL 3-necked round-bottomed flask, which was then sonicated using a Branson 1510 ultrasonicator (Branson Ultrasonics Corporation, Danbury, CT, USA) for 20 min while the flask was being purged with dry nitrogen gas to remove dissolved oxygen. After sonication and purging, the 3-necked round-bottomed flask was placed in a Rayonet photoreactor (Southern New England Ultraviolet Company, Branford, CT) equipped with G4T5 type mercury lamps and a cooling fan. The contents of the 3-necked round-bottomed flask were stirred using a paddle. The free radical photoreaction was performed at room temperature for 12 h using UV-A (315 nm to 400 nm) radiation.

**Figure 1:**
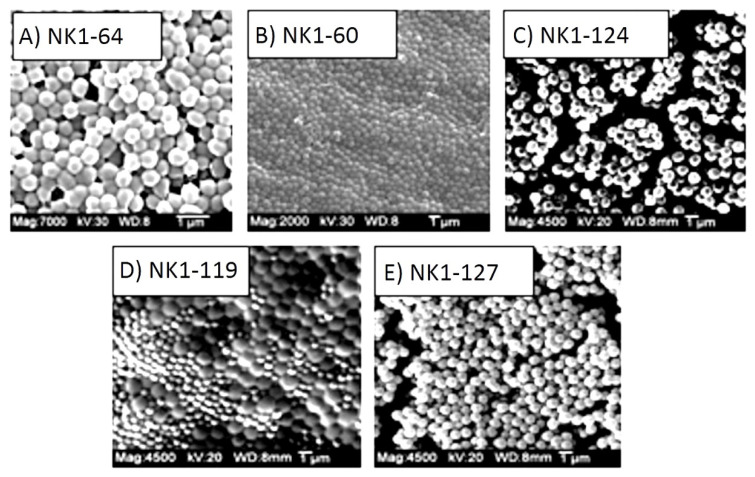
SEM snapshots of NIPA copolymers prepared by dispersion polymerization: (**A**) NK 1-64 (5% MAA), (**B**) NK 1-60 (10% MAA), (**C**) NK 1-124 (15% MAA), (**D**) NK 1-119 (20% MAA), (**E**) NK 1-127 (25% MAA).

After 12 h, the turbid polymer suspension was transferred into two 50 mL polypropylene centrifuge tubes and centrifuged at 3000 rpm for 10 min. After separating the supernatant, the particles were re-suspended in 25 mL aliquots of 90/10 (*v*/*v*) mixture of methanol and glacial acetic acid, sonicated for 30 minutes and centrifuged at 3000 rpm for 10 min. This washing procedure was repeated at least 4 times to remove unreacted monomer. Finally, the particles were washed 3 times with 25 mL aliquots of methanol, re-suspended in a small amount of methanol and stored in glass vials that were placed in a refrigerator until use. Scanning electron micrographs of polymer microspheres prepared, using this procedure, are shown in Figure 1.

### 2.4. Membrane Preparation

PVA hydrogel membranes were prepared by mixing the polyNIPA particles with aqueous PVA solution followed by crosslinking the PVA with glutaric dialdehyde. In a typical membrane preparation, 2 g of the mixture of polyNIPA microspheres (1%, *w*/*w*) dispersed in a PVA solution (8% *w*/*w*) was prepared in a 4 ml glass vial. The mixture was magnetically stirred overnight in order to homogeneously disperse the particles in the PVA solution. 50 μL of 10% aqueous glutaric dialdehyde was added using a micropipette and stirring continued for another hour. Finally, 50 μL of 1 M HCl was added as an initiator and after 2 min of stirring and sonicating the mixture was cast between two glass slides separated by 127 μm thick Teflon spacers. (The casting mold used for the immobilization membrane consisted of glass microscope slides edged with Teflon tape as a spacer on each of the long edges of the slide. A second microscope slide was used as a cover to create a cast of uniform thickness.) After a one-hour gelation period, the membrane reached its desired consistency and was separated from the slides and washed with plenty of DI water. Each membrane was inspected for uniformity by measuring its turbidity at every 5.0 mm and obvious irregular segments were removed. Generally, different segments from the same membrane gave the same results. All remaining segments for a membrane were stored in DI water in a sealed glass vial prior to use. Further details about the preparation of the PVA membranes and the measurements used to assess membrane uniformity can be found elsewhere [27].

### 2.5. Turbidity Measurements

Swelling and shrinking of the pH-sensitive polyNIPA particles embedded in the PVA membrane were investigated using a Cary 6000i double-beam spectrophotometer to measure turbidity. Each membrane was mounted on a custom built membrane holder placed in the sample cuvette. The membrane holder was constructed from black plastic to ensure that it did not pass stray light. Further details about the custom built membrane holders used and the mounting of the hydrogel membrane segment onto the holder can be found elsewhere [27]. 

The sample cuvette was fitted with a flow cell to allow for presentation of fresh sample solution (buffer of desired pH or DI water) to the PVA membrane containing the polyNIPA particles. The flow was regulated with a peristaltic pump at a rate of 1.0 mL/min. DI water was placed in the reference cuvette of the spectrometer. By changing the pH of the buffer solution in contact with the membrane, the pH response profile of the polyNIPA particles (embedded in a PVA membrane) was obtained. Although the absorbance spectra were collected over the wavelength range between 350 and 700 nm, all pH profiles (turbidity versus pH) were constructed using the absorbance data collected at 700 nm. By measuring the turbidity at longer wavelengths, the change in the refractive index of the membrane is the dominant optical effect as the intensity of the light scattered by the polymer decreases with increasing wavelength of the incident light.

For each turbidity measurement, the membrane was rinsed three times using a buffer solution of the prescribed pH. Although 90% of the pH response generally occurs within one minute, the membrane was allowed to equilibrate for 15 min prior to analysis by turbidity. Although the response time of the pH polymer particles is dependent on both the capacity of the buffer and the percentage of the 2-alkyl acrylic acid in the NIPA copolymer, 15 min was chosen to ensure that a full-scale response was obtained for each measurement. The rapid response of the membrane to the sample solution can be attributed to the porous nature of the PVA membrane, the size and shape of the pH-sensitive NIPA polymer microspheres, and the thickness of the PVA membrane which is approximately 127 μm. Colloidal-sized gel particles respond to external stimuli such as pH or temperature more quickly than bulk polymer films and as a result are more useful for chemical sensing.

## 3. Results and Discussion

### 3.1. Copolymers of NIPA and MAA

**Figure 2:**
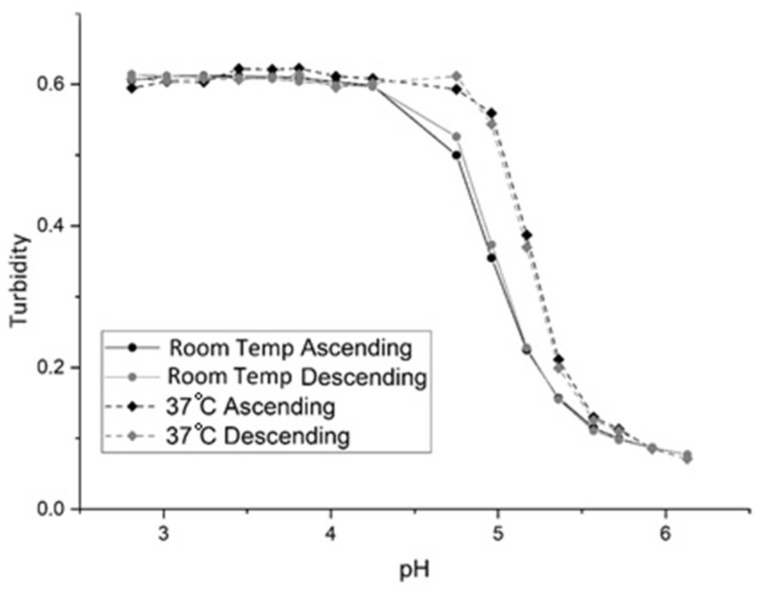
pH Response of NK 1-60 at room temperature and 37 °C for ascending and descending pH.

Figure 2 shows the pH response of NK 1-60 (see Table 1 for the formulation) from pH 3 to 7 (ascending) and pH 7 to 3 (descending) at 23 °C and 35 °C. Polymer swelling for NK 1-60 is reversible as the ascending and descending pH profiles are superimposable. At pH 3, the NK 1-60 particles exist in the shrunken state, whereas the particles exist in a swollen state at pH 7. In the shrunken state, the water content of the NK 1-60 particles is less than that of PVA, and the turbidity of the PVA membrane is large because the refractive index of the polymer particles is greater than that of PVA. In the swollen state, there is an increase in the water content and a decrease in the refractive index of the NK 1-60 particles. The PVA membrane is less turbid, reaching a limiting value that corresponds to the maximum swelling. (Although there is an increase in light scattering as the size of the NK 1-60 particles increases, it is the change in the refractive index of these particles that is the dominant effect.) For NIPA-based polymers such as NK 1-60, it is the polymer solvent interaction parameter [22] that governs swelling. As the pH of the buffer solution increases, the degree of ionization of the carboxylic acid group in MAA also increases. This, in turn, decreases the polymer solvent interaction parameter, causing the polymer to swell. Thus, pH-induced swelling and shrinking of NK 1-60 is controlled by changes in the polymer solvent interaction parameter.

The inflection point of the pH profile (that is, the point where the response is halfway between the response at low and high pH) is the apparent pK_a_ of the polymer. We use the term “apparent pK_a_” in this context to describe the inflection point as the relationship between turbidity and pH has not been addressed by theory in a manner that would allow the calculation of pK_a_ from the observed turbidity data. Since the change in the pH profile of the membrane occurs over a narrower pH range than a typical pH indicator (which is ±1 pH unit), it is likely that only partial deprotonation of the carboxylic acids occurs in order to achieve maximum swelling. As for the observed increase in pK_a_ with temperature (4.8 at 23 °C versus 5.2 at 35 °C, see Figure 2), this is the opposite of what occurs when a dye is used as the pH indicator in an optical fiber. The increase in the apparent pK_a_ of the polymer particles with temperature can be attributed to a decrease in the water content of the NK 1-60 particles. In all likelihood, there is a decrease in the distance between adjacent MAA units causing the deprotonation of MAA (which triggers polymer swelling) to occur at higher pH values.

**Figure 3:**
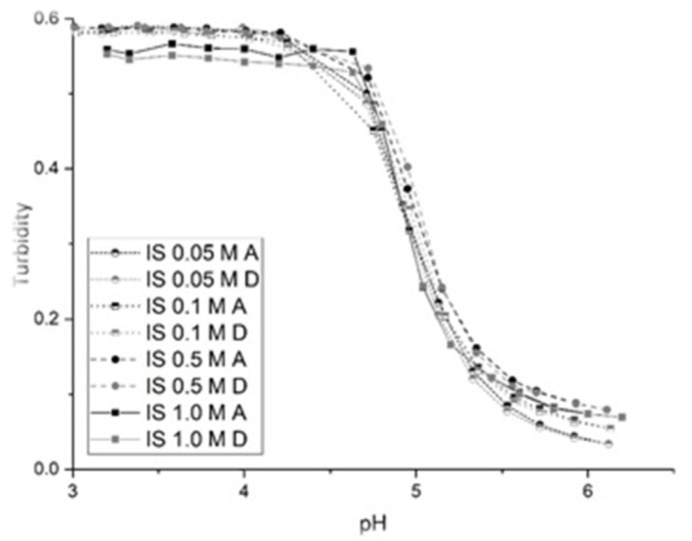
pH response profiles of NK 1-60 at the following ionic strengths: 0.05, 0.1, 0.5, and 1.0 M (black/A = ascending, gray/D = descending).

The swelling behavior of NK 1-60 was also investigated in both low and high ionic strength buffer solutions to gauge the effects of ionic strength on pH-induced polymer swelling. The NK 1-60 membrane test segment was exposed to buffer solutions of increasing ionic strength (IS). pH profiles were collected at 0.05, 0.1, 0.2, 0.5 and 1.0 M IS over the pH range 3.0–6.6 at 0.2 increments starting at pH 3.0. Figure 3 shows the pH profile at each of the five ionic strengths tested. There are no observable differences in the total swelling response nor are there significant differences in the overall shape and the location of the inflection points in these profiles. For NK 1-60, swelling is nonionic as the swelling behavior of NK 1-60 does not change at the ionic strengths surveyed (0.05 to 1.0 M). Furthermore, polymer swelling is reversible in the different ionic strength solutions surveyed as the ascending and descending profiles are superimposable.

**Figure 4:**
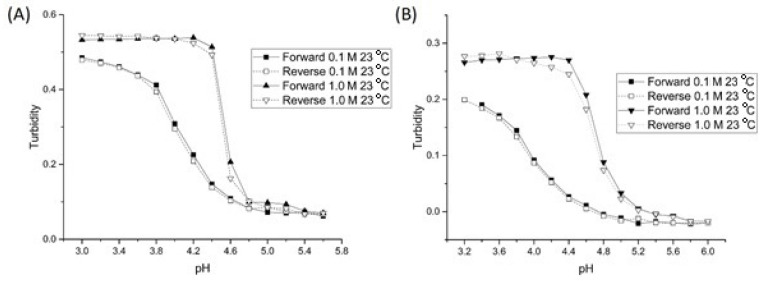
pH response curves of (**A**) NK 1-28 and (**B**) NK 1-77 at 0.1 and 1.0 M ionic strength.

Figure 4 shows the pH response curves of NK 1-28 and NK 1-77 (see Table 1 for the polymer formulation of each crosslinked NIPA copolymer) at 0.1 and 1.0 M IS. For each of the two crosslinked NIPA copolymers, there is an increase in the apparent pK_a_ as the ionic strength is increased (see Table 1). For these two copolymers, we attribute this increase to the penetration of chloride anions from the 1.0 M IS buffer into the polymer network of the NK 1-28 and NK 1-77 particles, thereby increasing the amount of chloride anions on or in the vicinity of the polymer backbone. This, in turn, increases the amount of negative charge in the proximity of the carboxylic acid groups. The result is an increase in the apparent pK_a_ of NK 1-28 and NK 1-77. Because of how the buffers were prepared (NaCl is added to the buffer to control their ionic strength), the increase in the apparent pK_a_ can also be viewed as a salt effect [22]. When the degree of crosslinking between the polymer chains is increased (e.g., NK 1-60), this effect (penetration of the chloride anions into the polymer network) is mitigated (see Figure 3). By adjusting the amount of crosslinker (MBA) present in the formulation, the response of the crosslinked NIPA copolymers can be tuned to yield pH profiles that are dependent (NK 1-28 and NK 1-77) or independent (NK 1-60) of the ionic strength of the buffer solution in contact with the membrane.

**Figure 5:**
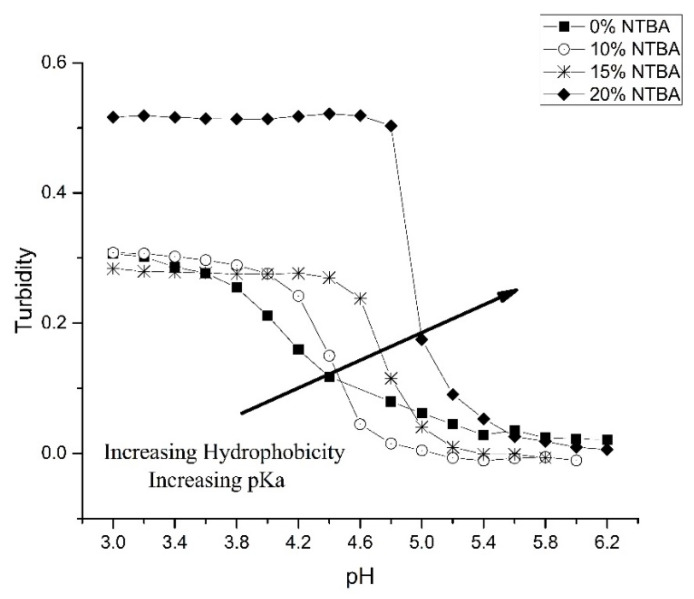
The effect of NTBA on the pH response profiles of several crosslinked copolymers of NIPA and MAA (NK 1-72, NK 1-77, NK 1-56, and NK 1-50).

Figure 5 shows the effect of the hydrophobic monomer NTBA on the pH response profiles of several crosslinked copolymers of NIPA and MAA (NK 1-72, NK 1-77, NK 1-56, and NK 1-50). Increasing the amount of NTBA in the formulation results in an increase in the apparent pKa of these copolymers (see Table 2). Swelling is also reversible as the ascending and descending pH profiles (not shown in Figure 5) are superimposable. Copolymerization of NIPA with NTBA [28] is known to decrease the lower critical solution temperature of polyNIPA. The increase in the apparent pK_a_ of this series of copolymers with increasing NTBA content can be attributed either to an increase in the hydrophobicity of the polymer formulation or a reduction in the lower critical solution temperature of these polymers.

**Figure 6:**
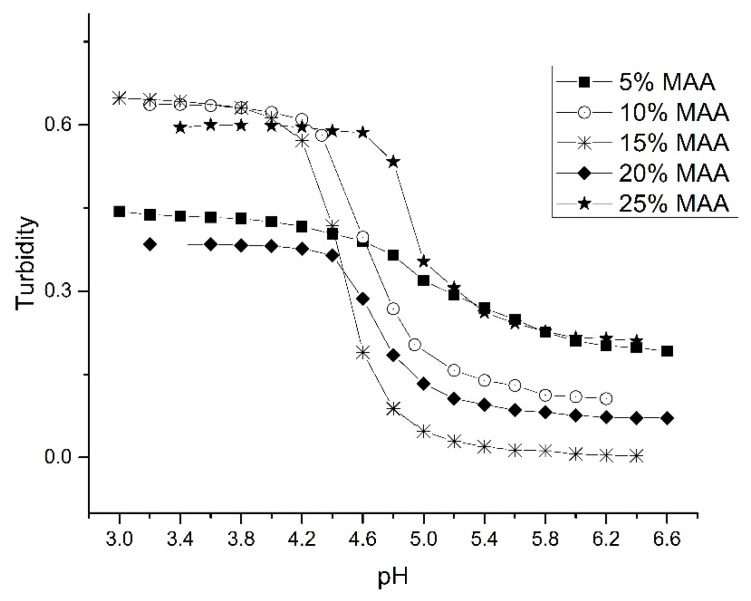
pH response profile for a series of NIPA–MAA copolymers prepared with increasing MAA content.

MAA plays a critical role in defining the pH response of the crosslinked NIPA copolymers. Acrylates (e.g., MBA, NTBA, and alkyl acrylic acids) are generally used as co-monomers because of their compatibility with NIPA. Alkyl acrylic acids (e.g., MAA) have a terminal carboxylic acid that contributes to polymer swelling through deprotonation. To determine the optimum amount of MAA in the formulation, the pH response of the particles was determined for a series of NIPA–MAA copolymers prepared with increasing MAA content (see Table 3). The pH profile of each of these copolymers is shown in Figure 6. The 5%, 10%, and 15% polyNIPA–MAA particles exhibit reversible swelling as their ascending and descending pH profiles (not shown in the figure) are superimposable. For this series of NIPA copolymers, the largest change in turbidity occurred when the amount of MAA present in the formulation was 10% or15%. Further increases in the amount of MAA resulted in a reduction in swelling. Therefore, the optimum amount of MAA in the formulation with respect to overall swelling is between 10% and 15%. For 5% MAA, there is a noticeable decrease in the overall swelling. However, lowering the percentage of MAA in the copolymer yields a product that responds over a wider pH range as well as shifting the apparent pK_a_ (see Table 3). The response time of the 5% MAA copolymer is also faster than the other copolymers in this series as a full-scale response occurred in less than five minutes. These results are consistent with the notion that only partial deprotonation of the carboxylic acid groups is sufficient to generate maximum swelling. The pK_a_ of the other NIPA copolymers (that contained higher amounts of MAA) varied from 4.5 for 15% MAA to 4.9 for 25% MAA (see Table 3).

### 3.2. Batch Variability

**Figure 7:**
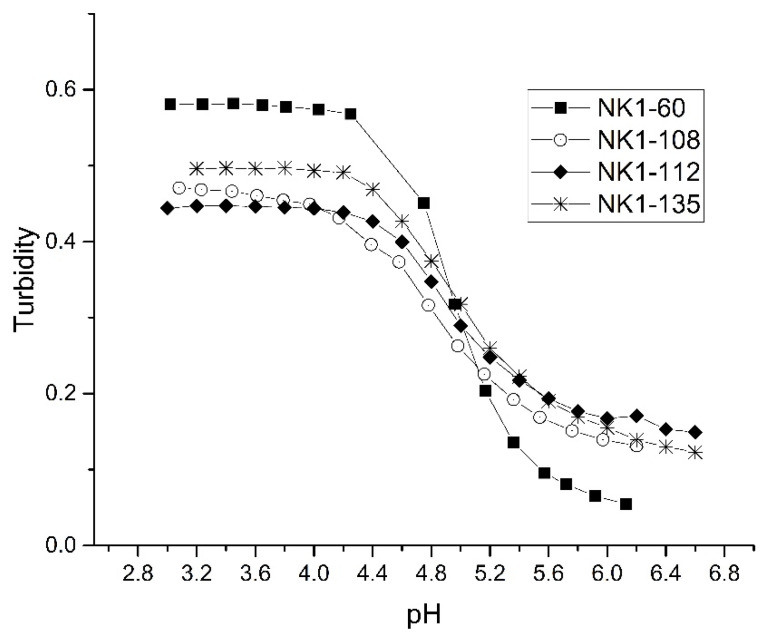
pH response profiles of four PVA membranes prepared from NK 1-60, NK 1-108, NK 1-112, and NK 1-135 polymer particles (70% NIPA, 10% MAA, 10% NTBA, 10% MBA).

Figure 7 shows a plot of the pH response profiles of four PVA membranes prepared from NK 1-60, NK 1-108, NK 1-112, or NK 1-135 polymer particles (70% NIPA, 10% MAA, 10% NTBA, and 10% MBA). This formulation was investigated because of the wealth of data available. For each batch of polymer particles prepared, the swelling was reversible as the ascending and descending pH profiles (not shown in the figure) are super-imposable. Although the “apparent pK_a_” is the same for all polymer particles, the total change in turbidity over the pH range investigated is not the same for all membranes (NK 1-60, NK 1-108, NK 1-112, and NK 1-135), indicating that only small differences exist among the crosslinked NIPA copolymers prepared using the NK 1-60 formulation. These results demonstrate that synthesizing microgels with reproducible swelling behavior is plausible even when the polymer formulation consists of several monomers.

### 3.3. Alkyl Acrylic Acids

Although it is only a minor component in the polyNIPA formulations investigated, MAA is able to impart the desired functionality to the microgel particles. Other functional co-monomers often reported in the literature [29] for NIPA are also from the acrylate family, wherein a terminal carboxylic acid is deprotonated as the pH of the solution in contact with the microgel particles is increased. A series of related acrylates (alkyl acrylic acids) were investigated to better understand how incorporating a series of pH-sensitive functional co-monomers of increasing hydrophobicity translates into higher pK_a_ values for the NIPA copolymer synthates. Carboxylic acid monomers selected for this phase of the study include AA, MAA, EAA, and PAA. Crosslinked NIPA copolymers were prepared using 10% AA, 10% MAA, 10% EAA, and 10% PAA (see Table 4).

**Figure 8:**
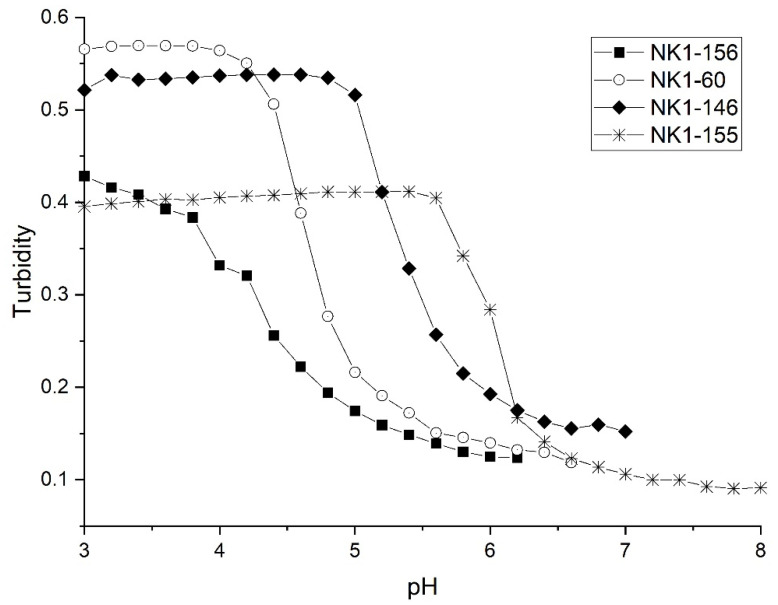
pH response profiles of four poly (*N*-isopropylacrylamides) synthates prepared by copolymerization of NIPA with AA (NK1-156), MAA (NK1-60), EAA (NK1-146), and PAA (NK1-155).

**Figure 9:**
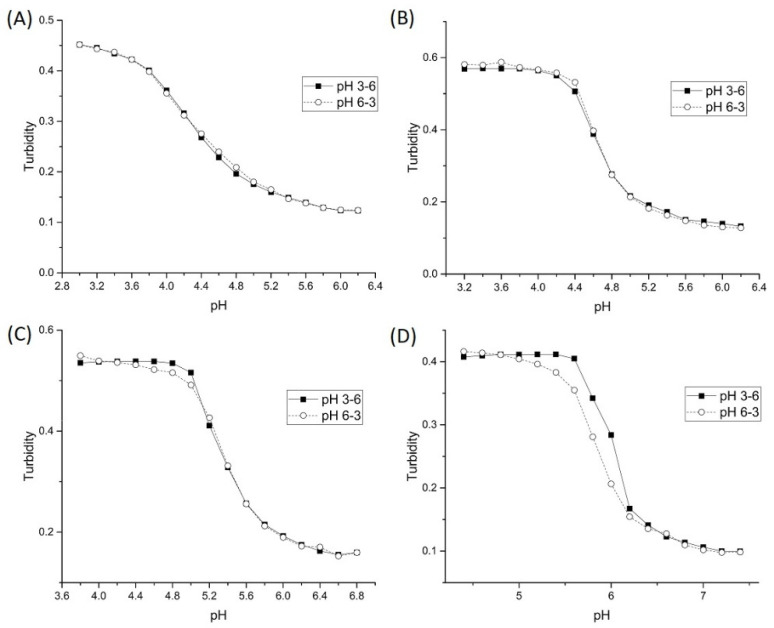
Forward and reverse ascending pH profiles of each polyNIPA synthate: (**A**) AA, (**B**) MAA, (**C**) EAA, and (**D**) PAA.

Figure 8 shows pH response profiles of four poly (*N*-isopropylacrylamide) synthates prepared by copolymerization of NIPA with AA, MAA, EAA, and PAA. Figure 9 shows the forward and reverse ascending pH profile of each synthate. Table 4 summarizes the formulations used, the apparent pK_a_ of each NIPA copolymer, and the pK_a_ of each functional comonomer. The AA functionalized copolymer has the lowest pK_a_ of the copolymers investigated in this series. A shift of 1.7 pH units in the apparent pK_a_ values are observed for the four copolymers in this series. Clearly, the apparent pK_a_ of these NIPA copolymers has been tuned by increasing the alkyl chain length of the carboxylic acid comonomer.

For the AA and MAA functional co-monomers, the apparent pK_a_ is in agreement with the pK_a_ of the free monomer (see Table 4), whereas the apparent pK_a_ of the NIPA copolymers of EAA and PAA is greater than the pK_a_ of the corresponding free carboxylic acid monomer. This can be attributed to the lower reactivity ratio of EAA and PAA. For all four NIPA copolymers, NIPA is the dominant monomer with only small quantities of the pH-sensitive functional comonomer present. When one monomer is present in greater amounts than another, longer chains containing the abundant monomer are observed with intermittent inclusion of the minor unit [30]. This is true if the relative reactivity ratio of all monomers is the same. For the case of AA and MAA, NIPA is polymerized into chains containing well separated functional comonomer units as the reactivity ratio of all monomers comprising each formulation is approximately the same. Because the reactivity ratio of EAA and PAA is lower [31], the corresponding NIPA copolymers do not contain well separated functional comonomer units resulting in higher apparent pK_a_ values. EAA and PAA are more closely oriented to each other than to what is observed for linear chains of NIPA and MAA and AA. The NIPA copolymers of EAA and PAA are more likely to form a block copolymer. 

To better understand the thermodynamics of pH-induced swelling of the four copolymers in this series, pH response curves were obtained at seven different temperatures to better understand the thermodynamics of pH-induced polymer swelling. The apparent pK_a_ was computed for each response curve and the relationship between the apparent pK_a_ and temperature was modeled using the van’t Hoff relationship, see Equation (1), where K_a_ is the apparent acid dissociation constant of the NIPA copolymer, *T* is the temperature, ∆*H*^0^ is the change in enthalpy for the pH-induced swelling of the poly (*N*-isopropylacrylamide) synthate, ∆*S*^0^ is the change in entropy associated with pH-induced swelling of the poly (*N*-isopropylacrylamide) synthate, and *R* is the gas constant.

(1) $$\ln\;K_{a}=\frac{-\Delta H^{0}}{RT}+\frac{\Delta S^{0}}{R}$$

Table 5 lists the changes in enthalpy and entropy that occur due to pH-induced swelling of the four poly (*N*-isopropylacrylamide) synthates. For AA, MAA, and EAA, the increase in the hydrophobicity of the pH-sensitive comonomer in the NIPA synthate can be correlated to an increase in the entropy and entropy of swelling. However, a decrease in the enthalpy and entropy of swelling for the copolymer of NIPA and PAA is observed. Furthermore, the pH-induced swelling behavior of the NIPA copolymers of AA, MAA, and EAA are reversible, whereas the pH-induced swelling behavior of the PAA copolymer is irreversible as the ascending and descending pH curves do not overlap (see Figure 9). In all likelihood, the PAA copolymer undergoes a conformational change when it swells and does not return to the same initial shrunken state. This would explain why PAA is a discordant data point in this series. 

It is the goal of this study to gain an understanding of how changes in the composition of the polymer formulation influence the pH response of these copolymers. By substituting AA, EAA, or PAA for MAA, we have shown how changes in the pH profile of the polymer particles (including shifts in the inflection point of the pH response curve) can be correlated to specific structural features of the crosslinked NIPA copolymers.

## 4. Conclusions

There are many advantages in using polymers for sensing applications. As polymers are composed of repetitive monomers, the substitution of these monomer units (e.g., alkyl acrylic acids) and the changes in their relative percent composition (e.g., MBA) can provide a nearly limitless reservoir of materials with customized properties. Copolymerization allows chemists versatility in creating materials with a wide variety of properties using a limited number of monomers. The ability to tailor the properties of materials that are readily prepared through free radical polymerization is paramount to meeting the specific requirements of sensing materials. Using pH-induced polymer swelling as an optical transduction mechanism ensures reversibility of the sensor response since swelling is reversible for most of the NIPA copolymers investigated. Additionally, the polymers can be coupled to fiber optic technology for remote sensing applications. 

## Figures and Tables

**Table 1:** Crosslinking Study.

NIPA Copolymer	*pK_a_ (0.1 M IS)	*pK_a_ (1.0 M IS)	NIPA	MAA	NTBA	MBA
NK 1-60 (10% crosslinking)	4.8	4.9	14 mmoles	2 mmoles	2 mmoles	2 mmoles
NK 1-28 (5% crosslinking)	3.9	4.5	17 mmoles	2 mmoles	0 mmoles	1 mmoles
NK 1-77 (5% crosslinking)	3.9	4.7	16 mmoles	1 mmoles	2 mmoles	1 mmoles

*pK_a_ of MAA monomer as determined by titration with NaOH standardized using KHP is 4.7.

**Table 2:** NTBA Study.

NIPA Copolymer	*pKa	NIPA	MAA	NTBA	MBA
NK 1-72 0% NTBA	4.10	16 mmoles	1 mmoles	0 mmoles	1 mmoles
NK 1-77 10% NTBA	4.43	15 mmoles	1 mmoles	2 mmoles	1 mmoles
NK 1-56 15% NTBA	4.73	14 mmoles	1 mmoles	3 mmoles	1 mmoles
NK 1-50 20% NTBA	4.91	14 mmoles	1 mmoles	4 mmoles	1 mmoles

*pK_a_ of MAA monomer as determined by titration with NaOH standardized using KHP is 4.7.

**Table 3:** Optimum Amount of MAA in Formulation.

NIPA Copolymer	pK_a_	NIPA	MAA	NTBA	MBA
NK 1-64 5% MAA	4.90	15 mmoles	1 mmoles	2 mmoles	2 mmoles
NK 1-60 10% MAA	4.57	14 mmoles	2 mmoles	2 mmoles	2 mmoles
NK 1-124 15% MAA	4.47	13 mmoles	3 mmoles	2 mmoles	2 mmoles
NK 1-119 20% MAA	4.66	12 mmoles	4 mmoles	2 mmoles	2 mmoles
NK 1-127 25% MAA	4.90	11 mmoles	5 mmoles	2 mmoles	2 mmoles

**Table 4:** Comparison of Alkyl Acrylic Acids.

NIPA Copolymer	Apparent pKa	pK_a_ of Functional Comonomer	NIPA	Functional Comonomer	NTBA	MBA
NK 1-156	4.37	4.2 (AA)	14 mmoles	2 mmoles of AA ^1^	2 mmoles	2 mmoles
NK 1-60	4.70	4.7 (MAA)	14 mmoles	2 mmoles of MAA ^2^	2 mmoles	2 mmoles
NK 1-146	5.39	4.7 (EAA)	14 mmoles	2 mmoles of EAA ^3^	2 mmoles	2 mmoles
NK 1-155	6.07	4.8 (PAA)	14 mmoles	2 mmoles of PAA ^4^	2 mmoles	2 mmoles

^1^ Log P value of acrylic acid is 0.29. ^2^ Log P value of methacrylic acid is 0.93. ^3^ Log P value of ethacrylic acid is 1.08. ^4^ Log P value of propacrylic acid is 1.59.

**Table 5:** Changes in Enthalpy ^1^ and Entropy ^1^ That Occur Due to Polymer Swelling.

Functional Comonomer	ΔH ^2^ (J/mol)	ΔS ^2^ (J/mol·K)
NK 1-156 (AA)	−47,000 ± 2300	−242 ± 7
NK 1-60 (MAA)	−74,900 ± 4400	−336 ± 15
NK 1-146 (EAA)	−100,000 ± 3300	−435 ± 11
NK 1-155 (PAA)	−60,000 ± 2400	−312 ± 8

^1^ Correlation coefficient of the least squares fit was greater than 0.99. ^2^ Uncertainties as determined by the linear least squares fitting of the data.

## Data Availability

The data presented in this study are available on request from the corresponding author.
